# The effects of shoulder arthroscopy on ultrasound image quality of the interscalene brachial plexus: a pre-procedure vs post-procedure comparative study

**DOI:** 10.1186/s12871-021-01409-3

**Published:** 2021-07-09

**Authors:** Jason K. Panchamia, Ram Jagannathan, Bridget P. Pulos, Adam W. Amundson, Joaquin Sanchez-Sotelo, David P. Martin, Hugh M. Smith

**Affiliations:** 1 Department of Anesthesiology and Perioperative Medicine, Mayo Clinic, 200 First St SW, Rochester, MN 55905 USA; 2 Department of Anesthesiology and Perioperative Medicine, Mayo Clinic Health System, Mankato, MN USA; 3 Department of Orthopedic Surgery, Mayo Clinic, Rochester, MN USA

**Keywords:** Brachial plexus block, Arthroscopy, Ultrasonography, Extravasation of diagnostic and therapeutic materials

## Abstract

**Background:**

Fluid extravasation from the shoulder compartment and subsequent absorption into adjacent soft tissue is a well-documented phenomenon in arthroscopic shoulder surgery. We aimed to determine if a qualitative difference in ultrasound imaging of the interscalene brachial plexus exists in relation to the timing of performing an interscalene nerve block (preoperative or postoperative).

**Methods:**

This single-center, prospective observational study compared pre- and postoperative interscalene brachial plexus ultrasound images of 29 patients undergoing shoulder arthroscopy using a pretest-posttest methodology where individual patients served as their own controls. Three fellowship-trained regional anesthesiologists evaluated image quality and confidence in performing a block for each ultrasound scan using a five-point Likert scale. The association of image quality with age, gender, BMI, duration of surgery, obstructive sleep apnea, and volume of arthroscopic irrigation fluid were analyzed as secondary outcomes.

**Results:**

Aggregate preoperative mean scores in quality of ultrasound visualization were higher than postoperative scores (preoperative 4.5 vs postoperative 3.8; *p* < .001), as was confidence in performing blockade based upon the imaging (preoperative 4.8 vs postoperative 4.2; *p* < .001). Larger BMI negatively affected visualization of the brachial plexus in the preoperative period (*p* < 0.05 for both weight categories). Patients with intermediate-high risk or confirmed obstructive sleep apnea had lower aggregate postoperative mean scores compared to the low-risk group for both ultrasound visualization (3.4 vs 4.0; *p* < .05) and confidence in block performance (3.8 vs 4.4; *p* < .05).

**Conclusion:**

Due to the potential reduction of ultrasound visualization of the interscalene brachial plexus after shoulder arthroscopy, we advocate for a preoperative interscalene nerve block when feasible.

**Trial registration:**

ClinicalTrials.gov (NCT03657173; September 4, 2018).

**Supplementary Information:**

The online version contains supplementary material available at 10.1186/s12871-021-01409-3.

## Background

Ultrasound guided interscalene nerve blockade (ISB) is considered the gold standard regional anesthetic technique for providing analgesia after shoulder arthroscopy, shoulder arthroplasty, and other upper extremity procedures [1, 2]. ISB can be performed before or after surgery; however, the optimal timing for ISB remains unknown. Practice patterns may be influenced by historical precedent, institutional protocols, and need for postoperative neurological assessment, among other considerations. Increased neck circumference and airway edema have been observed after arthroscopic shoulder procedures, suggesting extracapsular fluid extravasation and absorption into adjacent tissues may affect tissue planes of the neck where ISB is performed [3]. Complications of ISB, although rare, include major vascular injury, pneumothorax, brachial plexus palsy, nerve injury and phrenic nerve paresis. Thus, anatomical visualization of relevant structures is critical to its safe performance [4–6]. Considering implications of arthroscopic shoulder surgery - acute inflammation, muscle mobilization, and large volume irrigation - there is a deficiency of data regarding the impact of the arthroscopic surgery on ultrasound image quality during ISB. Subsequently, it is unknown whether the timing of the peripheral nerve block, with respect to the surgical procedure, impacts block efficacy and safety. Anecdotally, fellowship trained regional anesthesiologists at our institution who routinely perform ISB in different hospital settings, both before and after surgery, have observed that ultrasound image quality tends to be reduced in the postsurgical setting. Further, patients who do not receive preoperative blockade may be subject to increased perioperative opiate use, increased post anesthesia care unit (PACU) length of stay, and central sensitization to pain [7].

The objective of this study was to determine if a qualitative difference in ultrasound imaging at the interscalene level of the brachial plexus exists before and after arthroscopic shoulder surgery. We hypothesized expert regional anesthesiologists would consistently score preoperative ultrasound scans of the brachial plexus at the interscalene level higher in image quality and have greater confidence in performing ISB, in comparison to postoperative scans at the same level.

## Methods

We conducted a single-group, prospective observational study at a single academic hospital, using a pre-procedure vs post-procedure methodology where individuals served as their own controls. This study was approved by our Institutional Review Board (No. 18–004131, Mayo Clinic, Rochester, MN, USA) and was registered at ClinicalTrials.gov (NCT03657173). All patients provided written informed consent to participate, and the study protocol was performed in accordance with the relevant guidelines. The reporting of this study adheres to the Strengthening the Reporting of Observational studies in Epidemiology (STROBE) guidelines [8].

### Study patients

All adult patients (age ≥ 18 years) with an ASA physical classification of I to III scheduled for elective, arthroscopic shoulder surgery were recruited between June 2018 and November 2018. Patients were excluded if they fulfilled any of the following criteria: patient refusal to provide informed consent, cognitive disorder, allergy to study medications, BMI > 40 kg/m2, neck circumference > 50 cm, moderate to severe pulmonary disease (use of home oxygen, preoperative SpO2 < 94% on room air, forced expiratory volume in 1 s < 60% of predicted value), contralateral hemidiaphragm dysfunction, phrenic nerve injury, or a contraindication to regional anesthesia (neuropathy or coagulopathy).

Potential study subjects were identified from the operative calendar of three fellowship-trained orthopedic surgeons who practice at the same institution, use similar operative techniques, and perform a high volume of arthroscopic shoulder procedures. Study recruitment and informed consent occurred during preoperative consultation with the attending anesthesiologist, after the patient agreed to receive preoperative ISB and general anesthesia for an arthroscopic shoulder procedure. A member of the study’s research team then reviewed the informed consent documentation, corroborated inclusion/exclusion criteria, and enrolled those eligible for participation.

Patients were admitted to a preoperative procedure room 60 to 90 min prior to their scheduled surgery. Digital captures of a standardized ultrasound examination of the brachial plexus at the interscalene level were completed by three study personnel, all of whom were fellowship-trained regional anesthesiologists adept at ultrasound techniques. To standardize image capture, patients were placed in the supine position, with the head of the bed inclined 45 degrees, and the neck rotated toward the nonoperative side. After aseptic skin preparation with chlorhexidine and sterile draping, an ultrasound examination was performed using an X-Porte (SonoSite, Bothell, WA, USA) with a linear HFL38, 6- to 13-MHz ultrasound transducer, specifically observing the brachial plexus in the short-axis view. Images were captured in the following scanning sequence: (1) visualizing the brachial plexus at the supraclavicular level, (2) sliding the transducer proximally to the interscalene region to visualize the brachial plexus between anterior and middle scalene muscles, (3) sliding further proximally to view the cervical nerve roots entering neural foramina, and (4) sliding distally as the cervical nerve roots emerge between anterior and middle scalene muscles. Except for depth, which was adjusted to accommodate for patients’ body habitus as necessary, ultrasound settings were standardized to ensure uniform quality and image capture. After the initial ultrasound examination was completed, a 30 s prospective video clip was recorded using the aforementioned scanning procedure to better recreate real-time experience of ISB block performance.

After media acquisition, patients received a preoperative ISB as part of a standardized multimodal pain regimen focusing on preemptive analgesia and opioid reduction. The potential of residual local anesthetic and its impact on postoperative ultrasound imaging was examined considerably during study design. An extensive literature search revealed no published data demonstrating the visual identification of residual local anesthetic at set time intervals following regional blockade. Our collective observation, however, is that local anesthetic dissipates quickly following injection through mechanisms of absorption and dissemination through facial planes. Additional measures were implemented during ISB to minimize the possibility of residual local anesthetic during postoperative ultrasound imaging. Specifically, the same regional anesthesiologist involved with the ultrasound examination also performed the ISB at least 1 h prior to surgery using a single-injection, peri-plexus technique under continuous live ultrasound guidance; a 22-gauge 50 mm Stimuplex needle (B. Braun, Bethlehem, PA, USA) was advanced via an out-of-plane approach to the posterolateral border of the interscalene groove, located between the middle scalene muscle and brachial plexus fascial sheath in a single needle pass. A total volume of 5 to 10 mL of 0.5% bupivacaine with 1:200,000 epinephrine was administered while observing appropriate local anesthetic spread within the interscalene groove, and no hydrolocalization (repeated injections of solution to help navigate needle tip to target site) was used.

Following arthroscopic shoulder surgery, patients underwent postoperative ultrasound scanning within 15–30 min of arriving in the post anesthesia care unit, using the same standardized methodology. All media were extracted from the ultrasound machine via an encrypted USB flash-drive and edited to remove identification information including patient clinic number, date, and time. Each video file was renamed to a number, assigned by a random number generator, and placed in a password protected digital folder. Study personnel maintained a confidential record of the original media files with the corresponding file numbers.

**Fig. 1:**
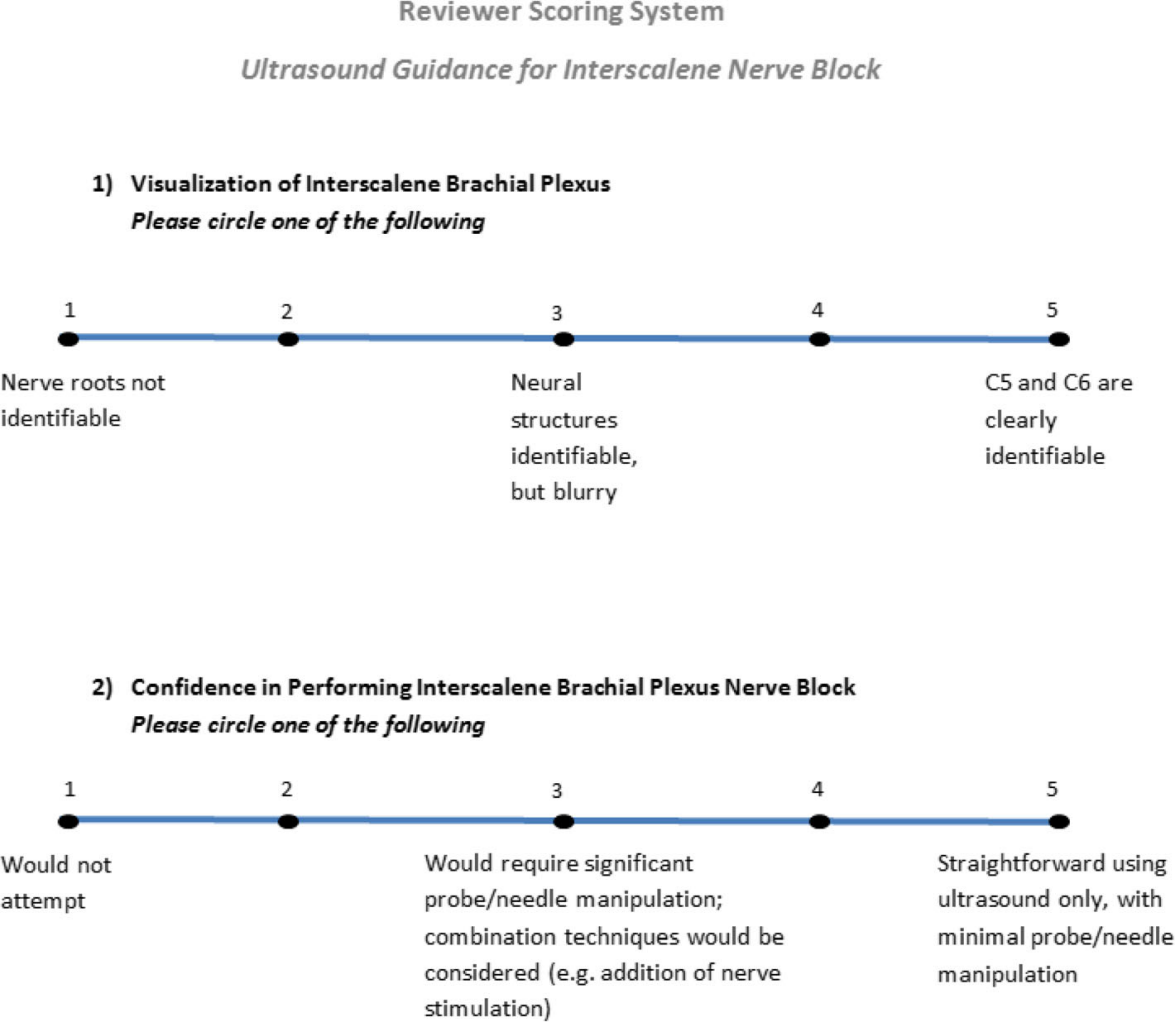
Reviewer scoring system for pre- and postoperative ultrasound examination using five-point Likert scale

Three fellowship-trained regional anesthesiologists with > 5 years of staff experience, who were not involved with the process of obtaining ultrasound images nor performed ISB for study patients, independently reviewed the ultrasound media on a high-quality desktop monitor (HP Elite Display E242, Hewlett-Packard, Palo Alto, CA, USA) in a private area. These expert reviewers were blinded to patient information, the image acquisition process, and the timing of when videos were obtained (pre- or postoperatively). Similar to the methodology by Neuts et al. [9] and Stolz et al. [10], the reviewers assessed visualization of the brachial plexus and their confidence in performing ISB, from the collected media, using a five-point Likert scale (Fig. 1). Reviewers could pause, fast-forward, and rewind video clips during review, without a set time limit.

### Outcomes

The objective of this study was to compare pre- versus post-surgical ultrasound imaging quality of the brachial plexus during arthroscopic shoulder surgery in patients receiving ISB. Primary outcomes evaluated include (a) observed differences in ultrasound image quality of the brachial plexus at the interscalene level and (b) differences in proceduralist’ confidence performing ISB, given this imaging.

Our secondary outcome was to evaluate surgical or patient variables that could influence pre- and postoperative ultrasound image quality. Factors analyzed included age, gender, BMI, duration of surgery, obstructive sleep apnea (OSA), and volume of arthroscopic irrigation fluid.

### Sample size

Since no prior data was available for the primary outcomes for this study, sample size calculations were performed using an effect size approach, where an effect size is defined as the difference in means divided by the standard deviation. The study was designed to have power to detect a medium effect size (i.e. an effect size of 0.5 to 0.8). Specifically, based on a paired t-test with alpha = 0.05 (two-sided), a power analysis demonstrated that a sample of 30 subjects would provide 80% power to detect an effect size of 0.50 or larger as being statistically significant. To accommodate attrition of approximately 10% (due to canceled surgery, patient dropout, and other reasons), a total sample size 33 patients was chosen.

### Data analysis

Data is reported using standard descriptive statistics, including means and standard deviations for continuous variables, and counts and percentages for categorical variables. The agreement between the 3 reviewers was analyzed using Fleiss’s Kappa statistic, and separate analyses were performed for each question for the preoperative data and the postoperative data. Comparisons of the reviewers’ ratings between the preoperative and postoperative periods were performed using Wilcoxon signed-rank tests; separate comparisons were performed for each reviewer, and for the average ratings of all 3 reviewers. The association of demographic and surgical factors with the reviewers’ ratings was analyzed using general linear models utilizing generalized estimating equations (GEE) to account for the within-subject correlation due to the study design in which each reviewer evaluated the same set of patient data. All statistical tests were two-sided and *p*-values less than 0.05 were considered significant. The analyses were conducted using SAS version 9.4 M6 (SAS Institute Inc., Cary, NC, USA) and R version 3.6.2 (R Foundation for Statistical Computing, Vienna, Austria).

## Results

Between June 2018 and November 2018, 33 patients undergoing elective, unilateral shoulder arthroscopy were enrolled into the study. Of the 33 patients, data from four patients were excluded due to conversion from arthroscopy to open surgical repair during the operation. A final sample size of 29 patients was used for data analysis, which according to our estimations provides 80% power to detect an effect size difference of 0.54 or larger (medium effect).

Demographics are displayed in Table 1. A majority of the patients underwent shoulder arthroscopy for rotator cuff repair (83%). The mean body mass index was 27.3 (Range: 20–37.9), and 11 of 29 patients had a diagnosis of OSA or were at intermediate to high risk for OSA per STOP-BANG criteria [11]. Average procedure duration was 81.2 min (Range: 24–162), and the mean irrigation volume used during shoulder arthroscopy was 21.5 l (Range: 12–60 l).

**Table 1:** Characteristics and surgical data of patients. Values are mean (SD) or number (proportion)

	Total (*N* = 29)
**Patient Age**	
Mean (SD)	59.7 (12.7)
Median	62.0
Range	23.0, 80.0
**Gender**, n (%)	
Female	13 (44.8%)
Male	16 (55.2%)
**BMI**	
Mean (SD)	27.3 (4.5)
Median	26.8
Range	20.0, 37.9
**BMI**, n (%)	
Normal (18.5–24.9)	10 (34.5%)
Overweight (25.0–29.9)	13 (44.8%)
Obese (≥ 30.0)	6 (20.7%)
**Diagnosis**, n (%)	
Rotator Cuff Tear	24 (82.8%)
Labral Tear	2 (6.9%)
Rotator cuff tendonitis	1 (3.4%)
Impingement	1 (3.4%)
Pectoralis minor syndrome	1 (3.4%)
**Side of Surgery**, n (%)	
Left	15 (51.7%)
Right	14 (48.3%)
**STOP-BANG Total Score**, n (%)	
0 to 2 (Low risk)	18 (62.1%)
3 to 4 (Intermediate Risk)	5 (17.2%)
≥ 5 (High risk)	1 (3.4%)
Confirmed diagnosis of obstructive sleep apnea	5 (17.2%)
**ASA score**, n (%)	
1	1 (3.4%)
2	23 (79.3%)
3	5 (17.2%)
**Number of irrigation bags**	
Mean (SD)	7.2 (3.7)
Median	6.0
Range	4.0, 20.0
**Total irrigation Vol (liters)**	
Mean (SD)	21.5 (11.0)
Median	18.0
Range	12.0, 60.0
**Procedure Duration (mins)**	
Mean (SD)	81.2 (37.7)
Median	72.0
Range	24.0, 162.0

Aggregate preoperative mean scores in quality of ultrasound visualization were significantly higher than postoperative scores (preoperative mean 4.5 vs postoperative mean 3.8; *p* < .001), as was confidence in performing blockade based on the preoperative study (preoperative mean 4.8 vs postoperative mean 4.2; *p* < .001) (Table 2). Comparisons of mean scores across the three reviewers demonstrate similar values with no statistical difference for pre- and postoperative quality of ultrasound visualization and confidence in performing ISB (Table 3). Inter-rater reliability displayed fair to good consistency among the reviewers for ultrasound brachial plexus visualization (pre-op: kappa 0.32, *p* < 0.001; post-op: kappa 0.41, *p* < 0.001), and confidence in block performance (pre-op: kappa 0.08, *p* = .45; post-op: kappa 0.50, *p* < 0.001).

**Table 2:** Individual reviewer and aggregate scores for pre- and postoperative ultrasound examination of brachial plexus

			Mean	Mean	Mean	
	Reviewer	N	Pre-op	Post-op	Difference	*p*-value
Quality of ultrasound visualization	1	29	4.4	3.7	−0.7	0.006
	2	29	4.7	3.9	−0.8	< 0.001
	3	29	4.5	3.8	−0.8	< 0.001
	Aggregate Mean	29	4.5	3.8	− 0.7	< 0.001
Confidence in performing ISB	1	29	4.8	4.1	−0.8	< 0.001
	2	29	4.9	4.2	−0.7	< 0.001
	3	29	4.8	4.3	−0.5	0.009
	Aggregate Mean	29	4.8	4.2	−0.7	< 0.001

*ISB* Interscalene nerve block

**Table 3:** Comparison across reviewers for pre- and postoperative quality of ultrasound visualization and confidence in performing ISB. Values are mean (SD)

		R1^a^ (*N* = 29)	R2^a^ (*N* = 29)	R3^a^ (*N* = 29)	Total (*N* = 87)	*p*-value
Quality of ultrasound visualization						
Pre-op	Mean (SD)	4.4 (0.7)	4.70 (0.5)	4.5 (0.5)	4.5 (0.6)	0.218
	Range	3.0–5.0	3.0–5.0	4.0–5.0	3.0–5.0	
Post-op	Mean (SD)	3.7 (0.9)	3.9 (0.8)	3.8 (0.9)	3.8 (0.9)	0.721
	Range	3.0–5.0	3.0–5.0	2.0–5.0	2.0–5.0	
Confidence in performing ISB						
Pre-op	Mean (SD)	4.8 (0.384)	4.9 (0.4)	4.8 (0.4)	4.8 (0.4)	0.444
	Range	4.0–5.0	3.0–5.0	4.0–5.0	3.0–5.0	
Post-op	Mean (SD)	4.1 (1.0)	4.2 (0.8)	4.3 (0.8)	4.2 (0.9)	0.657
	Range	3.0–5.0	3.0–5.0	3.0–5.0	3.0–5.0	

*ISB* Interscalene nerve block

^a^R1, R2, R3 refers to Reviewers 1, 2, and 3 respectively

Regression and subgroup analyses were performed for pre-defined variables which may impact ultrasound visualization and confidence in performing ISB (Table 4A, B, C, and D). High patient BMI negatively affected ultrasound visualization of the brachial plexus in the preoperative period (*p* < 0.05 for both weight categories; Table 4A). Patients with intermediate to high risk or confirmed OSA displayed lower aggregate postoperative mean scores in comparison to patients with low-risk OSA for both image quality (3.4 vs 4.0; *p* < .05) and confidence in performing ISB (3.8 vs 4.4; *p* < .05) (Table 5). Although the intermediate to high risk or confirmed OSA group had a larger difference in aggregate mean scores compared to low-risk OSA for both outcomes, this was not statistically significant.

**Table 4:** (A-D) Regression analysis of pre-defined variables during pre- and postoperative ultrasound examination

	Parameter estimate	*p*-value
A) Pre-op, ultrasound visualization		
(Intercept)	4.814	
Age	0.001	0.841
Female vs Male	−0.090	0.546
Obese vs Normal^a^	−0.524	0.022
Overweight vs Normal^a^	−0.433	0.009
B) Post-op, ultrasound visualization		
(Intercept)	4.458	
Age	0.003	0.772
Female vs Male	−0.236	0.431
Obese vs Normal^a^	−0.358	0.320
Overweight vs Normal^a^	−0.135	0.627
Duration (per 10 min)	−0.086	0.051
Fluid (per liter)	0.005	0.668
C) Pre-op, Confidence in ISB		
(Intercept)	5.186	
Age	−0.004	0.158
Female vs Male	−0.179	0.089
Obese vs Normal^a^	−0.293	0.140
Overweight vs ^a^ Normal	−0.017	0.800
D) Post-op, Confidence in ISB		
(Intercept)	5.666	
Age	−0.011	0.355
Female vs Male	−0.399	0.215
Obese vs Normal^a^	−0.405	0.372
Overweight vs ^a^ Normal	−0.276	0.385
Duration (per 10 min)	−0.051	0.329
Fluid (per liter)	−0.004	0.814

*ISB* Interscalene nerve block

^a^BMI classifications: Normal (18.5–24.9), Overweight (25.0–29.9), Obese (≥ 30.0)

**Table 5:** Sub-group analysis evaluating patients with low risk OSA and diagnosis of OSA or intermediate to high risk OSA

		Obstructive sleep apnea		
		Low-risk OSA (*N* = 18)	Intermediate/High risk/confirmed OSA (*N* = 11)	Low-risk vs Intermediate/High risk/confirmed OSA *p*-value^a^
**Quality of ultrasound visualization**	**Pre-Op**			
	Aggregate Mean (SD)^b^	4.6 (0.44)	4.5 (0.52)	0.642
	**Post-Op**			
	Aggregate Mean (SD)^b^	4.0 (0.70)	3.4 (0.71)	0.027
	**Pre-op vs Post-op** ***p*-value**	0.016	0.004	
	**Aggregate Mean Difference (SD)**	−0.5 (0.89)	−1.1 (0.76)	0.087
**Confidence in performing ISB**	**Pre-Op**			
	Aggregate Mean (SD)^b^	4.9 (0.16)	4.7 (0.42)	0.242
	**Post-Op**			
	Aggregate Mean (SD)^b^	4.4 (0.73)	3.8 (0.75)	0.028
	**Pre-op vs Post-op** ***p*-value**	0.016	0.004	
	**Aggregate Mean Difference (SD)**	−0.5 (0.72)	−1.0 (0.71)	0.076

*OSA* Obstructive Sleep Apnea, *ISB* Interscalene nerve block

^a^Unequal variance two sample t-test

^b^Aggregate mean of scores from Reviewers 1 to 3

## Discussion

The results of our investigation demonstrate that, in expert hands, ultrasound visualization of the brachial plexus and confidence in performing ISB was superior with imaging performed before surgery compared to the postoperative period in patients undergoing arthroscopic surgery of the shoulder. Though further studies are required, our results seem to indicate that the consistent use of preoperative ISB for shoulder arthroscopy may confer benefits of improved efficacy and safety when compared to postoperative block placement.

In addition, our findings show lower ultrasound image quality in obese patients compared to patients of lower body mass index, regardless of block timing. Numerous other investigations have similarly demonstrated poor visualization of peripheral nerves in obese patients, attributed to scattering of ultrasound waves in adipose tissue and greater depth required for imaging, which may compromise the sonographer’s ability to delineate the neurologic structures at the interscalene level [12–14]. Similarly, patients with OSA or those with intermediate-high risk of OSA had lower postoperative mean scores and larger differences in mean scores for both ultrasound visualization and confidence in block performance.

Although the results from our reviewers show a decrement in ultrasound imaging quality of the brachial plexus and decreased confidence in performing ISB, postoperative regression analysis did not show a statistical relationship to other secondary outcomes studied: age, gender, BMI, duration of surgery and volume of arthroscopic irrigation fluid. This lack of association with regard to irrigation fluid may be due to the sample size of the study, or that patients may vary widely in the degree of tissue extravasation, which may occur based on other demographic and/or surgical factors. Fluid extravasation from the shoulder compartment and subsequent absorption into adjacent soft tissue is a well-documented phenomenon in arthroscopic shoulder surgery. This has been associated with a number of possible complications, including severe head and neck swelling, airway compromise, prolonged intubation, and increased deltoid muscle compartment pressures from tissue edema [15–18]. Surgical risk factors accounting for fluid extravasation from the protective barrier of the glenohumeral capsule include prolonged procedure times, large volume of irrigation fluid, fluid pressures above 150 mmHg, use of automated pump systems, and anatomical abnormalities resulting in pathological tears or lesions in adjacent musculature [18, 19].

Consequently, mobilization of irrigation fluid between tissue planes in the head and neck may degrade ultrasound imaging of neurovascular structures, a phenomenon described as *layer non-differentiation*. Studies have demonstrated that tissue edema, leading to layer non-differentiation, had the highest odds ratio of predicting poor image quality amongst several studied artifacts known to compromise ultrasound imaging, which could explain our findings [20]. These risk factors may present a challenge for anesthesiologists attempting to stratify and predict patients with significant tissue edema postoperatively (the attached movie files show an ultrasound scan of the same patient before (Additional file 1) and after (Additional file 2) arthroscopic shoulder surgery. In “Additional file 2”, greater ultrasound depth was required to facilitate imaging of the brachial plexus due to tissue edema.

Previous investigations have highlighted numerous clinical advantages in performing ISB pre- versus postoperatively during shoulder arthroscopy. First, pre-emptive regional anesthesia techniques integrated into a multimodal analgesic pathway for orthopedic surgery have shown to improve perioperative outcomes, including reduced opioid consumption, improved hospital discharge times and increased patient satisfaction [7]. Moreover, use of preoperative blockade can provide intraoperative hemodynamic stability and improved surgical visualization without concomitant use of neuromuscular blockade under general anesthesia [21–23]. Lastly, the posterior cervical triangle contains important neurovascular structures (e.g. phrenic nerve, branches of subclavian artery) that are subject to potential injury during ISB and require optimal ultrasound imaging to improve block safety.

Considering all these factors in addition to our findings, preoperative performance of ISB for arthroscopic shoulder surgery may confer advantages in both efficacy and safety, since optimal ultrasound visualization enables providers to clearly differentiate neurologic targets from structures to be avoided. Certainly, we cannot draw direct conclusions among patients who experience clinically significant fluid extravasation and tissue edema, but it is logical that image quality degradation during an ultrasound-guided regional technique would decrease its efficacy and safety, especially over larger cohorts of patients in a common surgical procedure.

There are several limitations to this study. First, generalizability of our findings is limited to arthroscopic shoulder procedures; as such, the findings of our study cannot be extrapolated to other surgical procedures, such as shoulder arthroplasty. Second, we did not collect arthroscopic irrigation pressures, which could influence fluid extravasation postoperatively. However, this variable is difficult to collect accurately because the surgical team frequently adjusts pressures to maintain consistent field visualization. Third, residual local anesthetic injectate during postoperative ultrasound examination could be present. However, we strongly believe the likelihood is low for several reasons: a large interval between pre- and postoperative examination was intentionally devised to allow adequate time for local anesthetic absorption and fascial spread, a low volume of local anesthetic was utilized, and deposition of fluid adjacent to nerve roots create a tissue-fluid interface, thereby enhancing ultrasound visualization of neural tissue (sonographic accentuation of nerves against a hypoechoic background of fluid) [24–26], which consequently would improve reviewers’ postoperative ultrasound examination scores (contrary to our study findings). Lastly, convenience sampling and the subjective nature of the grading scales could increase the risk of bias. Although acquisition of the ultrasound images and video clips were standardized and blinded to the reviewers, these were recorded media files that relied on consistent ultrasound settings (depth, gain, etc.) from the proceduralist. This may impact the quality and interpretation of the brachial plexus imaging since reviewers did not perform dynamic ultrasound imaging. In addition, it is unknown if statistically significant differences in our results, comparing pre- vs. postoperative Likert scoring, are associated with clinically meaningful differences in block performance and success rates.

In conclusion, the effects of arthroscopic shoulder surgery on head and neck sonoanatomy should not be overlooked. The findings of our study demonstrate superior visualization and greater block confidence associated with preoperative ultrasound imaging of the interscalene brachial plexus in expert hands, compared to postoperative ultrasound imaging at the same level. This is particularly apparent in those with previously diagnosed OSA, or those who are intermediate-high risk of OSA per STOP-BANG stratification. Given the potential benefits of preoperative blockade on patient outcomes and pre-emptive multimodal analgesia, as well as the potential risks associated with postoperative blockade, we advocate for use of preoperative ISB for arthroscopic shoulder surgery when feasible. Further investigations are needed to corroborate these findings in a larger cohort and to identify patient and/or surgical-related factors, which may be modifiable, associated with tissue edema during arthroscopic shoulder surgery, specifically in practices where postoperative block performance is frequently utilized.

## Supplementary Information

**Additional file 1.** Preoperative Ultrasound Scan Video for Patient X. Description – ultrasound evaluation of interscalene brachial plexus prior to surgery displaying clear visualization of C5 and C5 nerve roots.

**Additional file 2.** Postoperative Ultrasound Scan Video for Patient X. Description – ultrasound evaluation of interscalene brachial plexus after shoulder arthroscopy displaying significant soft tissue edema and blurry visualization of C5 and C6 nerve roots.

## Data Availability

All data generated or analysed during this study are included in this published article.

## Abbreviations

ISB: Interscalene nerve block
PACU: Post-anesthesia care unit
STROBE: Strengthening the Reporting of Observational studies in Epidemiology
GEE: Generalized estimating equations
